# Liver Inflammation and Metabolic Signaling in Apc^Min/+^ Mice: The Role of Cachexia Progression

**DOI:** 10.1371/journal.pone.0119888

**Published:** 2015-03-19

**Authors:** Aditi A. Narsale, Reilly T. Enos, Melissa J. Puppa, Saurabh Chatterjee, E. Angela Murphy, Raja Fayad, Majorette O’ Pena, J. Larry Durstine, James A. Carson

**Affiliations:** 1 Integrative Muscle Biology Laboratory, Department of Exercise Science, University of South Carolina, Columbia, South Carolina, United States of America; 2 Center for Colon Cancer Research, Columbia, South Carolina, United States of America; 3 Division of Applied Physiology, Department of Exercise Science, University of South Carolina, Columbia, South Carolina, United States of America; 4 Department of Pathology, Microbiology & Immunology, School of Medicine, University of South Carolina, Columbia, South Carolina, United States of America; 5 Environmental Health and Disease Laboratory, Department of Environmental Health Sciences, University of South Carolina, South Carolina, United States of America; University of Louisville School of Medicine, UNITED STATES

## Abstract

The *Apc^Min/+^* mouse exhibits an intestinal tumor associated loss of muscle and fat that is accompanied by chronic inflammation, insulin resistance and hyperlipidemia. Since the liver governs systemic energy demands through regulation of glucose and lipid metabolism, it is likely that the liver is a pathological target of cachexia progression in the *Apc^Min/+^* mouse. The purpose of this study was to determine if cancer and the progression of cachexia affected liver endoplasmic reticulum (ER)-stress, inflammation, metabolism, and protein synthesis signaling. The effect of cancer (without cachexia) was examined in wild-type and weight-stable *Apc^Min/+^* mice. Cachexia progression was examined in weight-stable, pre-cachectic, and severely-cachectic *Apc^Min/+^* mice. Livers were analyzed for morphology, glycogen content, ER-stress, inflammation, and metabolic changes. Cancer induced hepatic expression of ER-stress markers BiP (binding immunoglobulin protein), IRE-1α (endoplasmic reticulum to nucleus signaling 1), and inflammatory intermediate STAT-3 (signal transducer and activator of transcription 3). While gluconeogenic enzyme phosphoenolpyruvate carboxykinase (PEPCK) mRNA expression was suppressed by cancer, glycogen content or protein synthesis signaling remained unaffected. Cachexia progression depleted liver glycogen content and increased mRNA expression of glycolytic enzyme PFK (phosphofrucktokinase) and gluconeogenic enzyme PEPCK. Cachexia progression further increased pSTAT-3 but suppressed p-65 and JNK (c-Jun NH2-terminal kinase) activation. Interestingly, progression of cachexia suppressed upstream ER-stress markers BiP and IRE-1α, while inducing its downstream target CHOP (DNA-damage inducible transcript 3). Cachectic mice exhibited a dysregulation of protein synthesis signaling, with an induction of p-mTOR (mechanistic target of rapamycin), despite a suppression of Akt (thymoma viral proto-oncogene 1) and S6 (ribosomal protein S6) phosphorylation. Thus, cancer induced ER-stress markers in the liver, however cachexia progression further deteriorated liver ER-stress, disrupted protein synthesis regulation and caused a differential inflammatory response related to STAT-3 and NF-κB (Nuclear factor—κB) signaling.

## Introduction

Cachexia is a wasting syndrome observed during the later stages of chronic diseases like cancer, Acquired Immunodeficiency Syndrome and Chronic Obstructive Pulmonary Disease [1], and greatly hampers quality of life in patients under remission. No pharmacological treatments are currently approved for cachexia [2]. This may be due to its multifactorial and systemic nature which could serve to limit the effectiveness of a single drug or therapy. It is therefore important to study the effect of cachexia progression not only in terms of loss of body mass, evident only in advanced stages of the disease, but also on initial systemic events that initiate and lead to wasting. Cachectic patients, along with an evident but gradual loss of fat and muscle mass, also manifest a host of underlying ailments such as chronic systemic inflammation, insulin resistance, increased gut permeability, anemia, anorexia, splenomegaly and disrupted metabolism [3–8]. Interestingly, the visceral organs such as heart, spleen, and liver maintain mass or even hypertrophy with cachexia [1].

Though chronic exposure to pro—inflammatory IL—6 has been reported to induce hyperplasia in the hepatic tissue, liver hypertrophy seen during cachexia is particularly intriguing [3,9], since nutrient depletion and increased energy demands induced by fasting [10] and infection depletes liver glycogen stores, which decreases liver mass [11,12]. In fact, liver hypertrophy is speculated to contribute to cachexia progression in cancer patients through the elevation of resting energy expenditure [3,13]. Liver governs the systemic metabolic rate by regulating pathways involved in utilization, transport, storage and breakdown of glucose and fat. Liver is also known to produce the acute phase proteins (APPs) in response to an inflammatory stimulus that can lead to degradation of muscle into amino acids [14,15]. Elevated pro—inflammatory cytokines during cachexia are known to initiate lipolysis [16], muscle wasting [17] and affect glucose metabolism [4,18]. Thus, chronic inflammation can increase metabolic demands and, coupled with inadequate nutrition, initiate rapid wasting. Since the liver has the potential to contribute to several wasting associated mechanisms, further research is needed to understand the role of the liver in cancer cachexia progression.

Current animal models used to study cachexia mimic varied subsets of the cachectic condition, and have provided evidence for the efficacy of treatments for the attenuation of muscle and fat loss. Recent studies with the C-26 tumor implant model of cachexia, have shown that cachexia induces an alteration in liver very low density lipid (VLDL) profile [19] and an induction of acute phase response in the muscle and the liver leading to muscle loss [14,15]. Tumor implantation models induce a rapid rate of weight loss; with mice losing around 20% of their body weight over a one week period [20], making it difficult to study mechanisms involved in cachexia progression. The *Apc* ^*Min*/+^mouse displays a sustained weight loss spanning approximately 6 weeks. While tumor development is initiated at 4 weeks of age [21] cachexia initiation is not seen until after 13 weeks of age and a severely cachectic phenotype is seen only at 18–20 weeks of age [6]. The gradual transition from a weight stable cancer state via a pre—cachectic to a severely cachectic state, is associated with plasma IL—6 levels and total tumor burden [22], making the *Apc* ^*Min*/+^ mouse an excellent model to study cachexia progression. Increasing tumor burden corresponds to increased levels of MCP-1 and IL—6 in the male *Apc* ^*Min*/+^ mouse [6,17,21,23]. IL—6 is also known to activate an APR in the liver and muscle, leading to secretion of APPs like haptoglobin and CRP, which further exacerbate the inflammation [7,15,24,25]. In addition, severely cachectic *Apc* ^*Min*/+^mice have elevated levels of serum endotoxin and increased gut permeability [6], which can also affect the liver. The purpose of this study was to determine if cancer and the progression of cachexia affected liver ER-stress, inflammation, metabolism, and protein synthesis signaling. We hypothesized that cachexia progression would increase liver inflammation leading to disruption of liver metabolic signaling and inhibit liver protein synthesis. In an effort to delineate the effect of cachexia from the effect of cancer, weight-stable *Apc* ^*Min/+*^ livers were compared to either wild-type livers (cancer effect) or to moderately and severely cachectic *Apc* ^*Min/+*^ livers (cachexia effect). Livers were analyzed for morphology, ER stress, glycogen content, inflammation, and metabolic changes.

## Materials and Methods

### Animals

All animal procedures were approved by the University of South Carolina’s Institutional Animal Care and Use Committee. Male *Apc* ^*Min*/+^ mice and C57BL/6 female mice were originally purchased from Jackson labs (Bar Harbor, ME, USA) and bred in the vivarium at the University of South Carolina. The initial litters were used to expand the breeding colony to obtain the animals required for the study. C57BL/6 and *Apc* ^*Min*/+^ mice were weaned at 3–4 weeks of age. The mice were group housed (maximum of 5 mice per cage) with mice of same age, sex and genotype being housed together. The mice were housed in a room kept at a 12:12hr light: dark cycle, with the light cycle starting at 07:00 hrs. The mice had *ad libitum* access to food (standard chow—Harlan Teklad Rodent Diet, #8604) and water. 8 week old male C57BL/6 and *Apc* ^*Min*/+^ mice were introduced into the study and randomized into 3 groups with similar average body weight. The groups were monitored for body weight loss and body temperature throughout the course of the study. As previously published, the initiation of weight loss in the *Apc* ^*Min*/+^ mouse occured at 13 weeks of age [21]. The weight of 12-week-old *Apc* ^*Min*/+^ mice was comparable to a healthy, age—matched C57BL/6 control [6]. Cachexia was initiated at 13–14 weeks of age with pre—cachectic mice exhibiting a significant weight loss that was less than 5% compared to the WT animals. Severely cachectic *Apc* ^*Min*/+^demonstrated a 20% body weight loss [17,22]. Serial blood draws taken during the study show that compared to age—matched C57BL/6 mice, the *Apc* ^*Min*/+^ mice are hyperlipidemic by 15 weeks and develop insulin resistance by 20 weeks of age [6].

Following an overnight fast, mice were sacrificed at 12 weeks (non—cachectic, N = 6), 14 weeks (pre—cachectic N = 6) and 18–20 weeks (severely cachectic, N = 6). Overnight fasting in animals controlled for the last eating bout, which helped reduce variation for markers related to protein synthesis measurements. Comparison between the WT and non—cachectic group highlights the effect of cancer, while comparisons between the *Apc* ^*Min*/+^ groups will tease out the effect of cachexia progression from the effect of cancer in these mice.

### Tissue collection

Mice were anesthetized using a ketamine cocktail, during the light cycle. The ketamine cocktail allowed for blood perfusion during until tissue collection and thus minimized tissue degradation during sacrifice. Plasma was collected prior to tissue collection via blood draws through the retro-orbital sinus. Liver was harvested during the sacrifice and was snap frozen in liquid nitrogen and stored at -80°C [17]. Intestine segments were isolated and cleaned. The small intestine was cut into 4 equal parts, and along with the colon was cut open vertically on a whatman filter paper and preserved using formalin. These were used to account for tumor burden in the cachectic *Apc* ^*Min*/+^ mice [6,17].

### RNA isolation and PCR

RNA extraction, cDNA preparation and real—time PCR was performed as described previously [26]. Briefly, RNA was isolated by homogenizing the liver tissue in Trizol (Invitrogen, Cat # 15596), followed by a chloroform/isopropyl alcohol extraction. cDNA was synthesized using the High Capacity cDNA Reverse Transcription Kit (Applied Biosystems, NY, USA) and RT-PCR assays were performed using the SYBR Select Master Mix (Applied Biosystems, NY, USA). Primers for SOCS-3 [15], Haptoglobin [15], PFK [27] and PEPCK [27] primers purchased from Integrated DNA technologies (Coralville, IA, USA). GAPDH was used as the housekeeping gene to normalize all the data obtained. A dilution curve for the samples was run at the starting of the study using GAPDH [22] to ascertain sample quality. Data was analyzed using the comparative cycle threshold [Ct] method calculated by the Applied Biosystems software.

### Western Blot

Western blots were performed as described previously [28]. Briefly, a piece of the liver was cut, weighed and placed in 10 times the volume of 1X Muller Buffer (50mM Hepes, pH 7.4, 0.1% TritonX—100, 4mM EGTA, 10mM EDTA, 15mM sodium pyrophosphate and 100mM β—glycerophosphate) [29]. The tissue was homogenized on ice, in the buffer using a glass on glass homogenizer. The resultant homogenate was quantified for protein concentration using the Bradford assay [21]. All protein samples were diluted to 3ug/ul concentration to aid equal loading on the gel. 15–60ug of protein was loaded on the gel to probe for proteins of interest. Homogenates were fractionated on SDS—polyacrylamide gels (6%- 15%) and transferred overnight onto a PVDF membrane. The membrane was stained with Ponceau to visualize an aberrations in protein loading. The PVDF membrane was then probed for phospho and total—STAT-3 (Ser 727), mTOR, S6 (Ser 235/236), Akt (Thr 308), p65 (S-468), MMP-2, IRE—α, phospho ERK (Thr 302/Tyr 204) and JNK (Thr 183/Tyr 185), and total Bip, CHOP (Cell Signaling Technology, Danvers, MA, USA) and GAPDH, Albumin, gp130 (Santa Cruz Biotechnology, Dallas, TX, USA) ATF6p50 and p-IRE-1α (Novus Biologicals, Littleton, CO, USA) A corresponding secondary antibody was used along with the chemiluminescent agent Quantum ECL (BioExpress, Kaysville, UT, USA) to visualize the protein bands. ImageJ (NIH, Bethesda, MD, USA) software was used for quantification of the integrated optical density (IOD) for Western blot bands.

### Hematoxylin and Eosin Staining

H&E stained sections were used to examine liver morphology. The pathological score for the sections was determined using the Histology Activity Index [30][31,32], by blinding the observer. Briefly, a subset of non—cachextic (N = 4) and severely cachectic (N = 5) *Apc* ^*Min*/+^ mice were perfused with 4% paraformaldehyde in PBS. The liver was stored in 4% paraformaldehyde overnight and transferred to a 30% sucrose solution. The perfused liver was mounted in a wax block and 4 μm sections were cut using a microtome. The sections were deparaffinized, stained with Hematoxylin and Eosin, and dehydrated using alcohol grades. Slides were mounted in the Permount media and imaged using the DP-70 camera.

### Periodic Acid Schiff’s (PAS) staining

Liver Glycogen was analyzed through the use of the PAS staining on liver cryosections [33]. C57BL/6 (N = 8), non—cachectic *Apc* ^*Min*/+^ (N = 5) and pre and severely cachectic *Apc* ^*Min*/+^ (N = 7) mice were used for this analysis. Briefly, a small piece of liver tissue was mounted on an OCT block and sectioned at a thickness of 10μm at -16°C. The slides were fixed in Carnoy’s fixative for 10 minutes followed by 30 minute incubation in the Periodic Acid. Slides were then washed with water and exposed to Schiff’s reagent for 30 minutes. The slides were counter stained with Hematoxylin, dehydrated through alcohol grades and mounted using Permount. The slides were imaged the next day using the DP70 Olympus microscope at a magnification of 200X. ImageJ was used to count the stained vs unstained pixels in each section. The ratio of the PAS stained area to the total area was determined and expressed as percentage for statistical analysis [34].

### Statistical Analysis

All statistical analysis was performed using the GraphPad Prism software (GraphPad, CA, USA). A One—Way ANOVA was performed to calculate the effect of cachexia with time in *Apc* ^*Min*/+^ mice. Post—Hoc Analysis was performed using the Student-Newman-Keuls test. A pre-planned t-test was performed to determine the effect of genotype—*Apc* ^*Min*/+^ as compared to WT animals. Liver glycogen content, as determined by PAS staining, was analyzed using the non—parametric Krushal—Wallis test. Significance was set at p<0.05.

## Results

The livers examined in this study were taken from *Apc* ^*Min*/+^ mouse classified as non—cachectic, pre—cachectic and severely cachectic mice as described in the methods section.

### Liver morphology during cachexia progression

**Fig 1:**
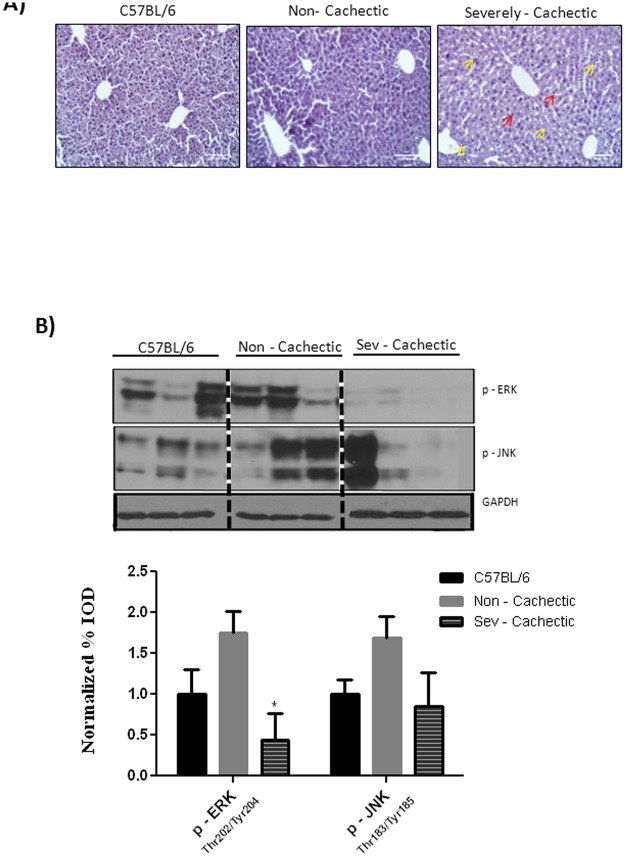
Effect of cachexia progression on liver morphology and MAPK signaling. A) Hematoxlyin and Eosin Staining of liver section for C57BL/6 (N = 3), Non—cachectic (N = 4) and severely cachectic (N = 4) *Apc* ^*Min*/+^ mice. Pathological scoring for the sections was done in accordance to the HAI scale B) Expression of levels of phosphorylated ERK and JNK in the liver (N = 6 per group). Values are expressed as Mean ± SE. * denotes significantly different from the non—cachectic *Apc* ^*Min*/+^ mouse analyzed by One—Way ANOVA. p < 0.05.

A subset of WT, non—cachectic and severely—cachectic mice were perfused using 4% paraformaldehyde fixative and stained with the hematoxylin and eosin stain to determine if cachexia progression leads to liver pathology. As determined by the Histology Activity Index, non—cachectic livers showed very few (shown by red arrows) bipolar nuclei with some liver injury concentrated near the central vein or acinar zone 3 areas (Fig. 1A). As opposed to cancer only (non—cachectic) livers, severely cachectic livers displayed signs of mild to moderate liver injury with signs of liver regeneration along with minimal scarring, and infiltrating liver leukocytes as shown by yellow arrows (Inflammation score: 9–12 using histological activity index criteria); especially in the sinusoids as compared to the C57BL/6 mice (Fig. 1A). Protein expression of the mitotic marker ERK showed no significant difference in the non-cachectic mice but was inhibited in severely cachectic mice. On the other hand, the inflammatory and stress marker JNK was variable and showed no change with cachexia progression in the *Apc* ^*Min*/+^ mouse (Fig. 1B).

### The Effect of Cancer on Liver Signaling and Gene Expression

**Fig 2:**
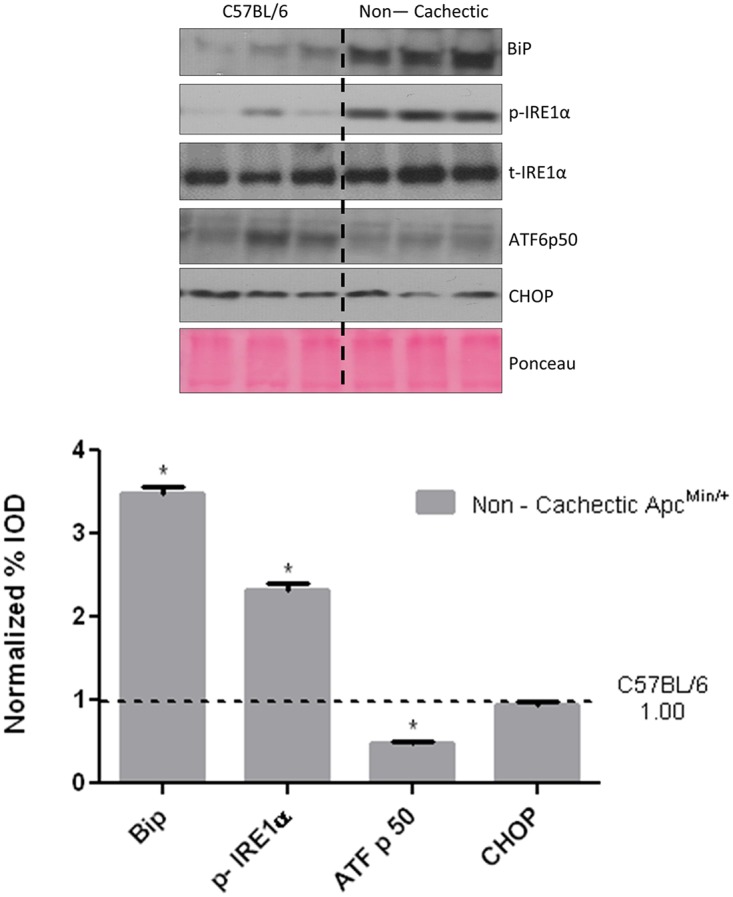
Effect of cancer on ER stress markers. Bip1, IRE-1, ATF-6 p50 and CHOP expression in the liver of non—cachectic *Apc* ^*Min*/+^ mice (N = 6 per group), compared to healthy C57BL/6 mice. Dotted line on the western blot indicates two different sections of the same gel. Values are expressed as Mean ± SE. * denotes significantly different from the healthy C57BL/6 mice as analyzed by a pre—planned t—test. p < 0.05.

**Fig 3:**
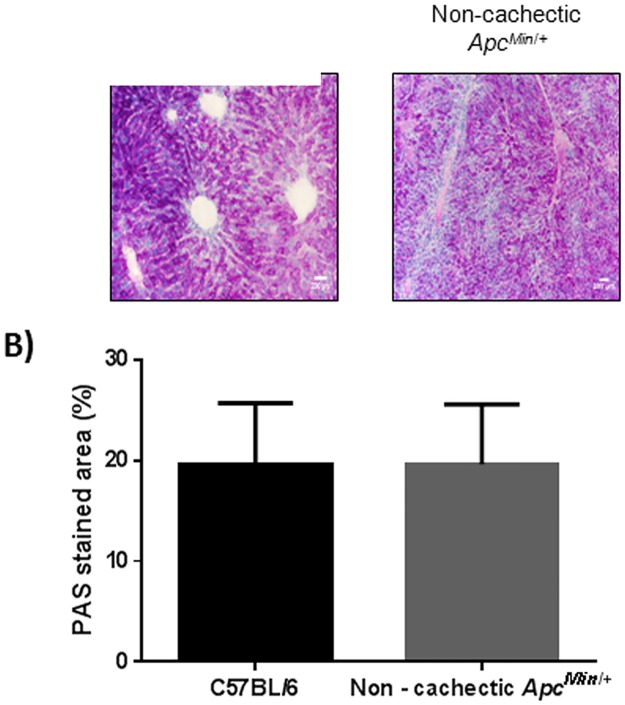
Effect of cancer liver glycogen stores. A) Glycogen stores as determined by PAS staining. B) Morphometry for the PAS stain to estimate glycogen stores in the WT and non—cachectic liver. N = 8 for healthy C57BL/6 and 5—non—cachectic *Apc* ^*Min*/+^ were used for the analysis. Values are expressed as Mean ± SE. p < 0.05.

**Fig 4:**
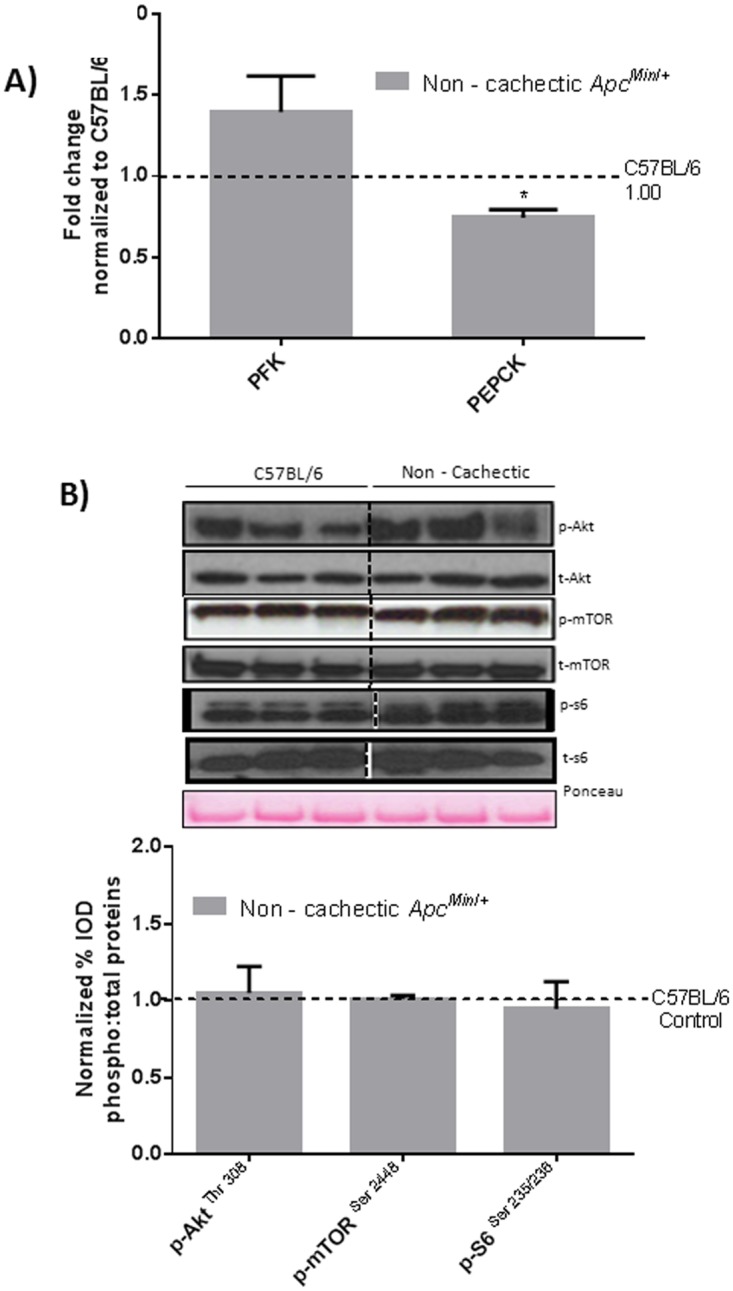
Effect of cancer on liver metabolic and anabolic signaling in non—cachectic mice. A) Liver mRNA expression of metabolic genes PFK and PEPCK B) Protein expression liver anabolic signaling in the non-cachectic mice. Values are expressed as Mean ± SE. * denotes significantly different from C57BL/6 as analyzed by a pre—planned t—test. Values are normalized either to the respective total protein for phosphoproteins and to GAPDH for non—phosphorylated proteins. (n = 5–6 per group, p < 0.05) Dotted line on the graph indicates levels of C57BL/6, while the dotted line on the western blots indicate two different parts of the same gel.

**Fig 5:**
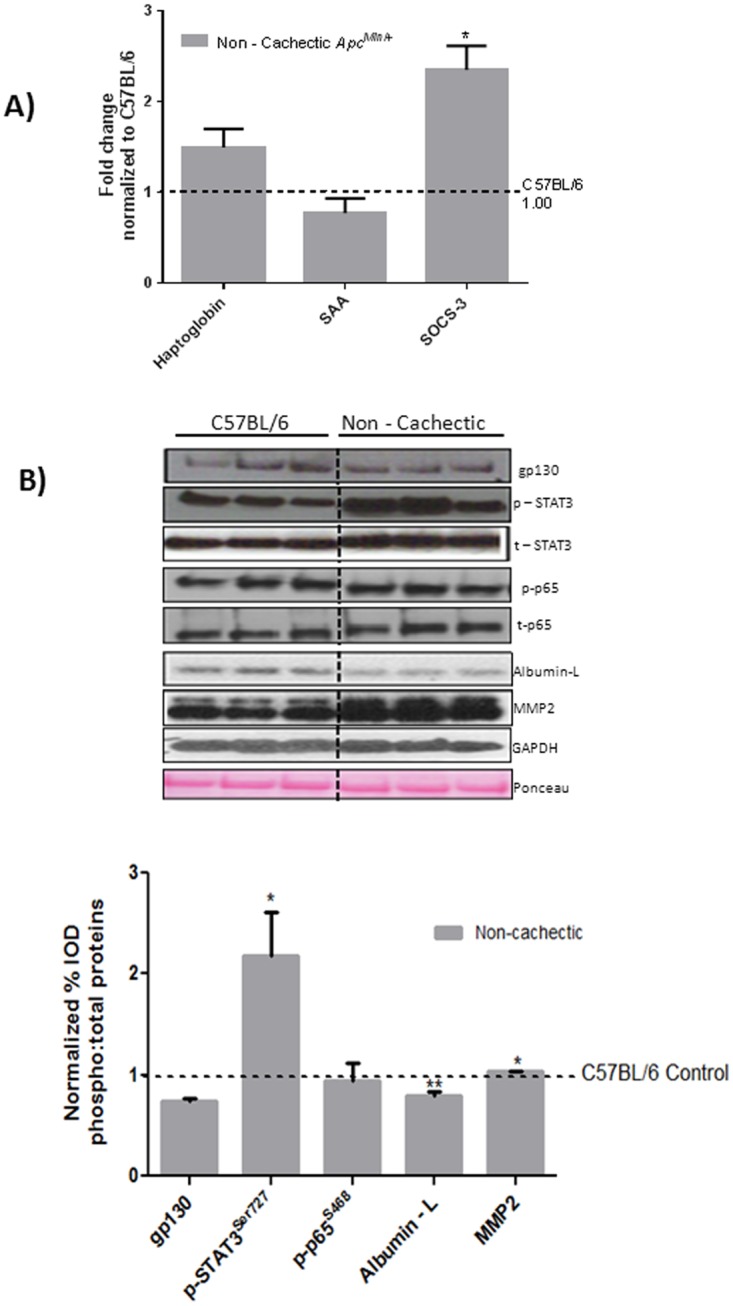
Effect of cancer on liver inflammatory signaling in non—cachectic mice. A) Liver mRNA expression of inflammatory markers B) Protein expression liver inflammatory signaling in the non-cachectic mice. Values are expressed as Mean ± SE. * denotes significantly different from C57BL/6 as analyzed by a pre—planned t-test. Values are normalized either to the respective total protein for phosphoproteins and to GAPDH for non—phosphorylated proteins. (n = 5–6 per group, p < 0.05) Dotted line on the graph indicates levels of C57BL/6, while the dotted line on the western blot indicates two different parts of the same gel.

ER stress signaling in the liver was examined in the WT and non—cachectic *Apc* ^*Min*/+^mice. The expression of the unfolded protein chaperone—Bip/GRP78, and the ER stress transducers IRE-1, ATF6 and CHOP were examined. We report that cancer induced liver Bip/GRP78 and IRE1α while suppressing the expression of ATF6 (Fig. 2). Liver glycogen content was determined using PAS staining and quantified using morphometry. Non—cachectic mice did not show a change in liver glycogen content with the cancer (Fig. 3). We found no effect of cancer on PFK mRNA expression (Fig. 4A). However, PEPCK mRNA expression was significantly reduced by 45% with cancer (Fig. 4A). Phosphorylation of Akt, mTOR and S6 were unaffected by cancer in the non—cachectic mice as measured by western blot (Fig. 4B). Liver SOCS3 mRNA expression was induced by cancer in non-cachectic mice (Fig. 5A). The mRNA expression of APPs, haptoglobin and serum amyloid A, was not altered by cancer (Fig. 5A). Cancer increased liver STAT-3 phosphorylation approximately 2-fold (Fig. 5B), which coincided with a significant 20% reduction (p = 0.002) in liver albumin protein concentration. There was a small, but significant increase in liver MMP2 protein expression (Fig. 5B). Cancer did not change liver glycoprotein 130 (IL-6β receptor) expression or phosphorylated p65 protein expression (Fig. 5B). These results demonstrate that cancer induces liver STAT-3 signaling with a corresponding increase in SOCS3 mRNA expression.

### The Effect of Cachexia Progression on Liver Signaling and Gene Expression

**Fig 6:**
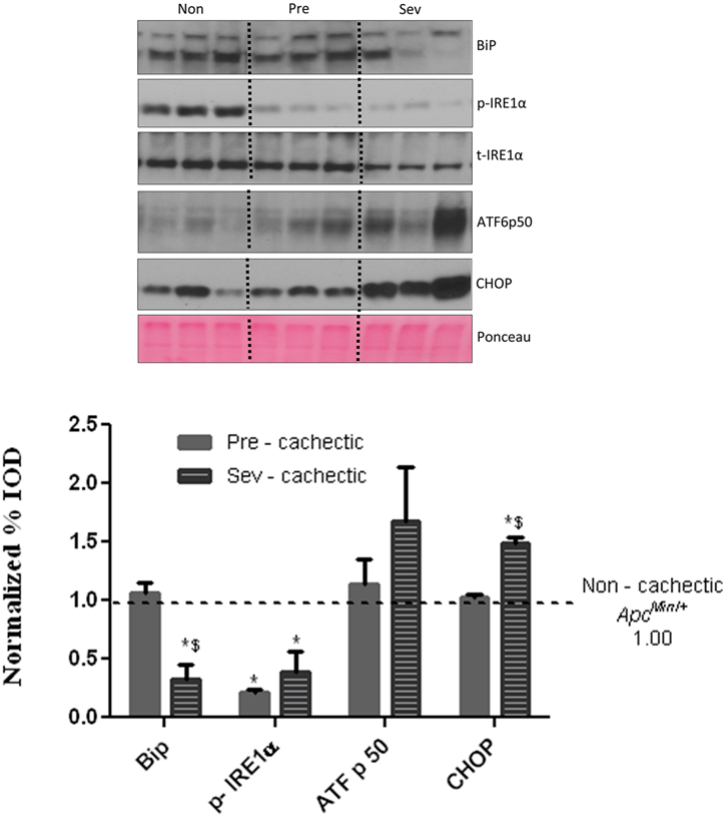
Hepatic ER stress markers with cachexia progression. ER stress markers Bip, IRE1α, ATF6p50 and CHOP were examined in the liver of non, pre and severely cachectic mice. Values are expressed as Mean ± SE. (n = 6–8 per group, p < 0.05) Dotted line indicates levels of Non—cachectic mice. Non = Non—Cachectic *Apc* ^*Min*/+^ Sev = severely cachectic *Apc* ^*Min*/+^; * denotes significantly different from Non—cachectic *Apc* ^*Min*/+^ $ denotes different from the pre—cachectic *Apc* ^*Min*/+^ mice, as analyzed by a One—Way ANOVA, p < 0.05.

**Fig 7:**
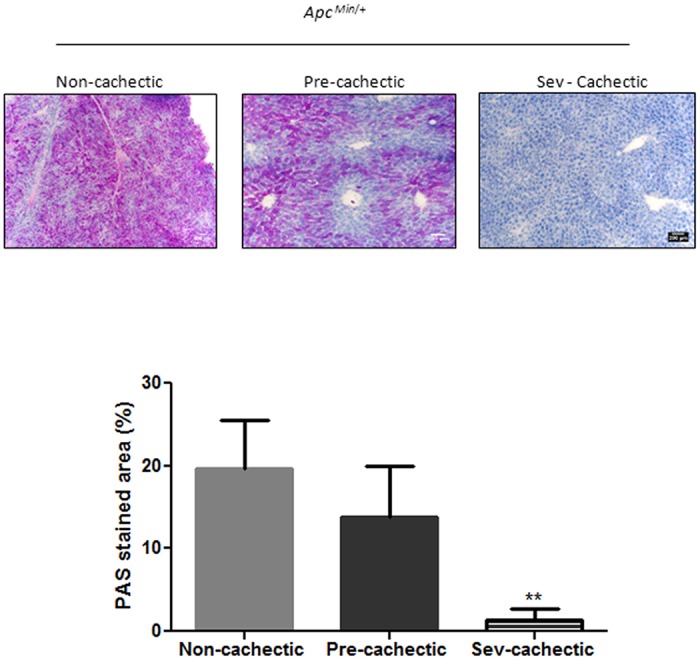
Changes in liver glycogen stores with cachexia progression. Glycogen stores as determined by PAS staining and quantified using the ImageJ software in the non (N = 5), pre (N = 7) and severely (N = 7) cachectic *Apc* ^*Min*/+^ mice. Values are expressed as Mean ± SE. * denotes significantly different from the Non—cachectic *Apc* ^*Min*/+^ as determined by One—Way ANOVA, p< 0.05.

**Fig 8:**
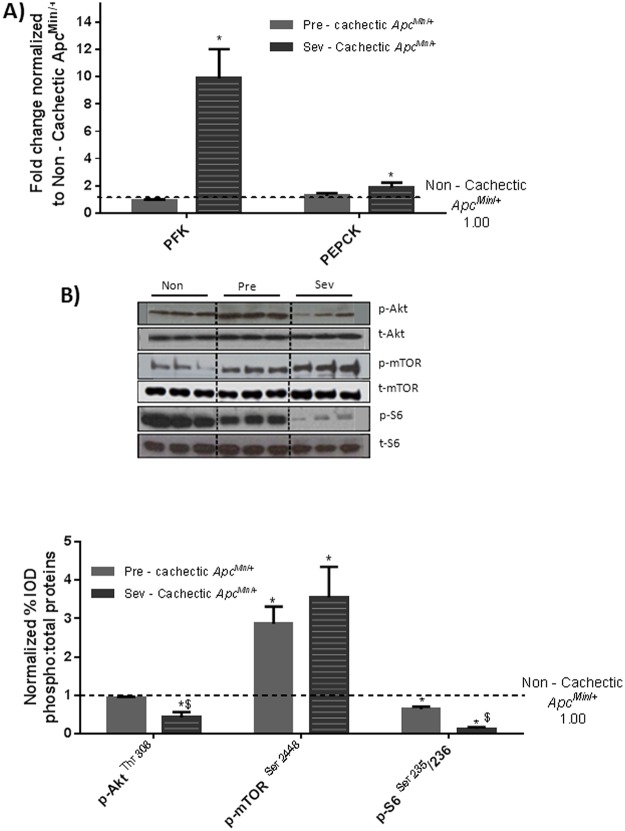
Changes in liver metabolic and anabolic markers with cachexia progression. A) Liver mRNA expression of metabolic genes PFK and PEPCK B) Protein expression liver anabolic signaling with cachexia progression. Values are expressed as Mean ± SE. * denotes significantly different from Non—cachectic *Apc* ^*Min*/+^ $ denotes significant difference from the pre—cachectic *Apc* ^*Min*/+^ mice as analyzed by One—Way ANOVA. Values are normalized either to the respective total protein for phosphoproteins and to GAPDH for non—phosphorylated proteins. (n = 5–6 per group, p < 0.05) Dotted line on the graph indicates levels of Non—cachectic *Apc* ^*Min*/+^, while a dotted line on the Western blot indicates different regions of the same gel. Non = Non—Cachectic *Apc* ^*Min*/+^ Sev = severely cachectic *Apc* ^*Min/+*^.

**Fig 9:**
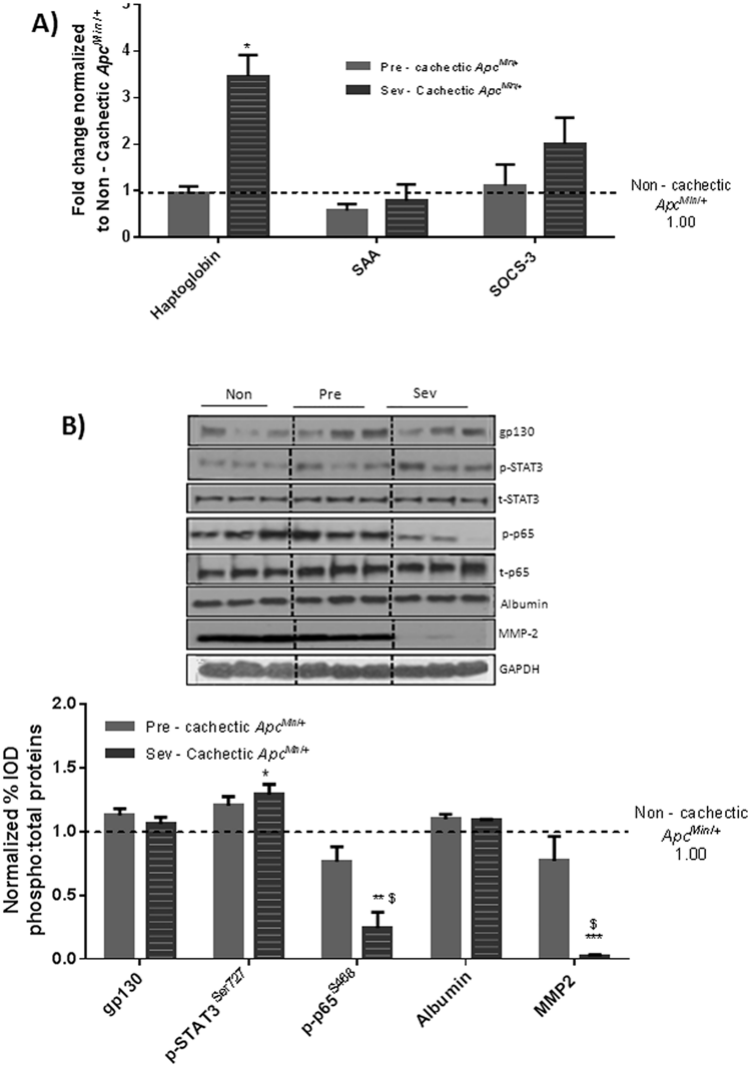
Liver inflammatory signaling with cachexia progression. A) Liver mRNA expression inflammatory markers B) Protein expression liver inflammatory signaling with cachexia progression. Values are expressed as Mean ± SE. * denotes significantly different from Non—cachectic *Apc* ^*Min*/+^ $ denotes different from the pre—cachectic *Apc* ^*Min*/+^ mice, as analyzed by One—Way ANOVA. Values are normalized either to the respective total protein for phosphoproteins and to GAPDH for non—phosphorylated proteins. (n = 5–6 per group, p < 0.05) Dotted line indicates levels of Non—cachectic *Apc* ^*Min*/+^. Abbreviations: Non = Non—Cachectic *Apc* ^*Min*/+^ Pre = Pre-cachectic *Apc* ^*Min*/+^ Sev = severely cachectic *Apc* ^*Min/+*^.

To examine the effect of cancer cachexia progression we examined non-cachectic, pre-cachectic, and severely cachectic *Apc* ^*Min*/+^ mice. Cachexia progression suppressed Bip/GRP78 and p-IRE-1α expression, but there was no further effect on ATF6p50 expression. Expression of the apoptotic marker CHOP was induced in the severely cachectic mice, which coincided with Bip/GRP78 and IRE1α suppression (Fig. 6). Liver glycogen content was depleted in the severely cachectic *Apc* ^*Min*/+^ mice as compared to the non—cachectic and the pre—cachectic mice (Fig. 7). The progression of cachexia induced liver PFK mRNA expression 11-fold and PEPCK mRNA expression 2-fold (Fig. 8A). No difference in either PFK or PEPCK gene expression was observed early in cachexia, as pre—cachectic mice were not different from non—cachectic mice. A significant inhibition of liver Akt and S6 phosphorylation was observed with cachexia progression. Interestingly, mTOR phosphorylation was increased both in the pre—cachectic and severely cachectic *Apc* ^*Min*/+^ mice (Fig. 8C). SOCS3 expression did not change further with cachexia progression (Fig. 9a). Acute phase gene expression for haptoglobin was elevated ~3.5 fold, but SAA expression was not significantly different from the non—cachectic *Apc* ^*Min*/+^ mice (Fig. 9A). Liver haptoglobin expression was increased in livers from severely cachectic mice, but not in pre—cachectic mice. Cachexia progression further increased STAT-3 phosphorylation, though there was no change in liver gp130 and albumin protein content with cachexia progression (Fig. 9B). Interestingly, cachexia progression suppressed NF-κB phosphorylation ~ 75% in the severely cachectic mice as compared to non—cachectic mice and ~65% as compared to pre—cachectic mice (Fig. 9A). Liver MMP-2 expression, an angiogenic and fibrotic marker, was suppressed 90% in the severely cachectic mice (Fig. 9B).

## Discussion

Since it is likely that the liver is a pathological target of cachexia progression, our study under took the novel examination of the liver in a cancerous state combined with cachexia. We report that hepatic stress can be observed in the form of ER stress in the non—cachectic cancer mice, however significant disruption of liver inflammatory, metabolic and protein synthesis signaling were observed only with progression of cachexia. Livers from severely cachectic mice showed signs of leukocyte infiltration and mild injury that were accompanied by increased haptoglobin transcription. Additionally, indices of metabolic dysfunction were present in cachectic livers, as there was a depletion of glycogen and altered expression of the glycolytic enzyme, PFK, and the gluconeogenic enzyme, PEPCK. Liver Akt/mTOR/S6 regulation was also disrupted by cachexia. Cachexia suppressed liver Akt and S6 phosphorylation, independent of mTOR, which was induced with cachexia progression. Interestingly, in the cachectic liver, expression of the fibrosis and angiogenic marker, MMP 2, was suppressed along with NF-κB activation and MAPK phosphorylation. There was a corresponding increase in the ER stress induced apoptotic marker CHOP. Thus, cachexia progression disrupted several indices of liver signaling and gene expression and further work is needed to establish their role in the overall wasting process.

While intestinal and colon tumor burden has been shown to be directly associated with cachexia development in *Apc* ^*Min*/+^ mice, we have previously reported that non—cachectic *Apc* ^*Min*/+^ mice have a similar number of tumors as severely cachectic *Apc* ^*Min*/+^, but these tumors are smaller in diameter [6]. Thus, circulating factors related to the increased tumor burden, such as IL-6 and MCP-1, may have an important role in cachexia development. McClellan *et*. *al* have reported increasing levels of plasma MCP—1 levels in *Apc* ^*Min*/+^ mouse starting as early as 8 weeks of age. Plasma MCP-1 is known to activate the zinc finger protein MCPIP (MCP- 1 inducible protein) that can lead to induction of ER stress [23,35]. Correspondingly, we report an increase in ER stress markers in the non—cachectic *Apc* ^*Min*/+^ mouse, likely indicating problems in hepatic protein folding. Unlike MCP-1, serum IL—6 levels are not elevated in non—cachectic *Apc* ^*Min*/+^ mice [22] and no change was seen in the levels of the downstream gp130 receptor protein expression in the liver. The hepatic APR was not induced with cancer alone, as liver haptoglobin levels were comparable to the healthy C57BL/6 mice. IL—10 and other IL—6 family cytokines like LIF, OSM, IL-11 are known to be elevated in the plasma of some implant cachexia models [15,36] and though the presence of these cytokines has not been established in the *Apc* ^*Min*/+^ mouse, there is a possibility that these could play a role in STAT-3 activation in the non—cachectic mice. Increased SOCS3 at this stage could be a downstream response to increased STAT-3 signaling. Interestingly, inhibition of IL—6 signaling can induce liver fibrosis by induction of MMP-2 [37]. The slight induction of MMP—2 expression by cancer could possibly be the result of SOCS3 mediated IL—6 pathway inhibition.

The complexity of cachexia regulation is demonstrated by the decrease in body mass, attributed to loss of fat and muscle, while other organs such as the spleen and liver hypertrophy. Liver hypertrophy combined with the cancer-induced suppression of gluconeogenic signaling could indicate a metabolic disruption that involves glycogen utilization. Interestingly, liver glycogen levels were depleted in cachectic mice, but not in weight stable mice with cancer. The cachexia-induced loss of liver glycogen was accompanied by increased PFK and PEPCK gene expression and could indicate increased glucose flux related to the cachectic metabolic state. An acute inflammatory response can inhibit proteins synthesis and deplete liver glycogen as seen during pathogen-induced inflammation and starvation experiments [11,12]. In fact, IL—6 infusion in vivo has been shown to induce hepatic hyperplasia, independent of hepatic growth factor activation in the liver [9]. However, cancer alone did not alter liver protein synthesis regulation through—Akt-mTOR-S6. However, an increased tumor burden can induce the Warburg effect, increasing lactic acid concentrations in the cytosol [38], and subsequently converting it to glucose via Cori’s cycle in the liver [39,40]. Elevated glucose levels in the liver could be instrumental in hepatic PEPCK mRNA suppression observed in the weight stable mice as increased glucose—insulin signaling can act as a negative feedback for gluconeogenesis [41–44].

**Fig 10:**
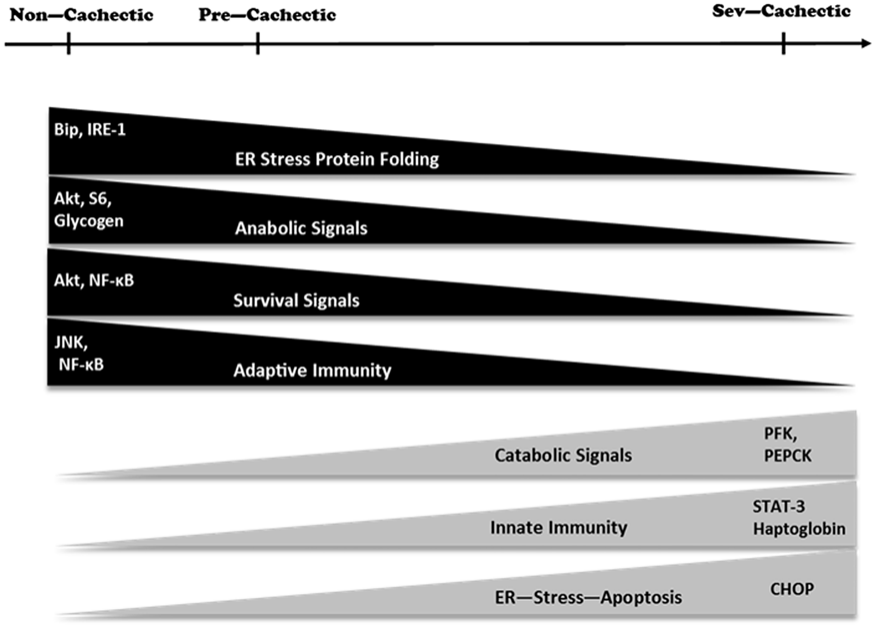
Schematic diagram describing the molecular signaling associated with cachexia progression in the liver.

While cancer cachexia progression is accompanied by chronic systemic inflammation, our examination of liver inflammation showed some very interesting and diverse developments (See Fig. 10). There was evidence of inflammation related to leukocyte infiltration and the induction of the APR in the liver, but surprisingly the activation of the classical NF-kB and JNK pathways were suppressed during severe cachexia. Moreover, no evidence of liver fibrosis was observed in the morphological analysis, reiterating a suppression of the immune response. Hepatic STAT-3 phosphorylation, however, is also considered to be an inflammatory marker and is sufficient to induce an IL-6 dependent muscle atrophy in the *Apc* ^*Min*/+^ mouse with cachexia progression [6,17,21]. Phosphorylated STAT-3 is also the major transcription factor responsible for the transcription of haptoglobin, the APP that was increased in the cachectic *Apc* ^*Min*/+^ liver [30]. However, apart from its early induction in the weight—stable cancer mice, no further increase was observed in SOCS-3 with cachexia progression, highlighting a disconnect between STAT-3 and its downstream negative regulator with chronic IL-6 signaling. Hepatic IL-6/STAT-3 signaling is responsible for the suppression of liver dendritic cells in an immature state, allowing for tolerance of toxins entering the liver through the portal vein. Secretion of IL-6 is induced in the liver via LPS from the gut bacteria and protects the liver from the production of TNF—α. Lack of IL- 6 causes liver DCs to produce higher levels of TNF—α which can lead to fibrosis. This is a liver defense mechanism which is known to elevate the threshold stimulus necessary to convert the innate triggers into an adaptive response [45,46]. IL—6 is known to be protective against liver fibrosis, with IL—6 knockout mice showing increased liver fibrosis and insulin resistance upon CCl4 administration [47]. There is the possibility that tumor secreted IL—6 in the *Apc* ^*Min*/+^ mouse protects the liver from an inflammatory and fibrotic reaction in the same manner. Thus, during cachexia progression, the hepatic inflammatory response seemed to be restricted to the innate arm, with a possible suppression of the adaptive immune responses.

However, hepatic MMP-2 inhibition with cachexia progression can also be attributed to corresponding p-65 inhibition. Since phosphorylation of NF- κB was inhibited in the cachectic *Apc* ^*Min*/+^ along with a suppression of Akt, this could provide evidence that for an apoptotic phenotype in the liver. NF-κB liver knockouts undergo apoptosis in the face of an immune and concavalin-A challenge [48,49]. Endotoxin levels are known to be elevated in the cachectic *Apc* ^*Min*/+^ sera, along with high circulating IL—6 levels. Thus, increased inflammatory response coupled with inhibition of the p-65 expression could trigger hepatocyte apoptosis in the *Apc* ^*Min*/+^ mice. Although the induction of the IL—6/STAT-3 pathway is known to be pro—survival, with activated STAT-3 blocking the effects of FAS activation [50], these beneficial effects are only observed with an acute bout of IL-6 [51]. Chronic exposures to IL—6 are in fact known to induce apoptosis and lead to liver failure [51]. The suppression of survival signals combined with altered Akt / mTOR signaling, could point towards an endoplasmic reticulum (ER) stress induced apoptosis. The ER stress response is regulated by three ER-localized proteins: ATF6, PERK, an upstream regulator of eIF2α, and IRE1α as well as various molecular chaperones, including BiP. These sensors are activated in order to bring homeostasis back to the cell under conditions in which there is a buildup of mis- and/or unfolded proteins. However, under chronic stress conditions in which the cell cannot cope with the multitude of improperly formed proteins, the downstream ER stress marker, CHOP, is upregulated leading to cell death. At the onset of cancer, in non-cachectic mice, we found an increase in BiP and p-IREα, with no change in the expression of CHOP, suggesting that the early stages of ER stress had commenced with cancer. With cachexia progression, we actually found both BiP and p-IRE1α to be suppressed, whereas CHOP content increased leading us to surmise that the hepatocytes had transitioned over to an apoptotic state resulting from the chronic cellular stress placed upon the cells [52–54]. With this being said, further research is needed to better understand the role that ER stress plays in the suppression of survival signaling in the cachectic liver.

Hepatic apoptosis could also explain the elevated plasma endotoxin levels in the severely cachectic *Apc* ^*Min*/+^ mice as the liver fails to filter out the excess endotoxin. Elevated systemic LPS and chemokines like MCP—1 levels are known to attract leukocytes to the affected area [55–57]. Severely cachectic mice had an infiltration of leukocytes in the liver, but this was not observed in the weight stable mice. However, MCP—1 levels are known to be elevated even in the non—cachectic cancer mice [6,23]. Thus it is possible that elevated levels of endotoxin direct leukocyte infiltration of the liver in the severely cachectic *Apc* ^*Min*/+^ mice.

## Conclusion

In conclusion, a causal relationship between cachexia progression and the deterioration of liver was readily apparent by the results of our study (see Fig. 10). As compared to non—cachectic mice with cancer, the liver in severely cachectic mice was under metabolic stress with depleted glycogen and altered metabolic gene expression. Additionally, liver Akt / mTOR signaling was disrupted by cachexia. Severely cachectic mice displayed a robust acute phase protein response to the elevated levels of IL6/STAT-3 signaling. The inhibition of Akt and NF-κB in the cachectic liver, along with the induction of ER stress could point to problems with cell survival with the progression of cachexia. Additional experiments need to be performed to establish a mechanistic link for the liver during cachexia progression, and should be pursued as a future line of inquiry for understanding the devastating consequences of cachexia.

## Supporting Information

S1 Arrive Checklist (PDF)
